# The Application of Genetic and Genomic Biotechnology in Aquaculture

**DOI:** 10.3390/biology12010127

**Published:** 2023-01-13

**Authors:** Baofeng Su, Xu Wang, Rex A. Dunham

**Affiliations:** 1 Alabama Agricultural Experiment Station, Auburn, AL 36849, USA; 2 School of Fisheries, Aquaculture and Aquatic Sciences, Auburn University, Auburn, AL 36849, USA; 3 Department of Pathobiology, College of Veterinary Medicine, Auburn University, Auburn, AL 36849, USA

This Special Issue, “The Application of Genetic and Genomic Biotechnology in Aquaculture,” collates 14 published manuscripts covering different aspects of implementing advanced molecular genetics and genomic science in aquaculture. The research interests included:

1. Gene-editing on the gonadotropin-releasing hormone (GnRH);
2. Sex-reversal of adult eastern mosquitofish;
3. MicroRNA in sex differentiation, circRNA in ovarian development and maturation, molecular network (i.e. mRNA–lncRNA–miRNA) in steroidogenesis and spermatogenesis;
4. Alternative splicing in salinity adaptation and eyestalk translocation;
5. Identification of antimicrobial peptide genes and the immune function of cirRNA–miRNA–mRNA in response to bacterial infection;
6. Hypoxia-induced pathways;
7. Genome resequencing for population genomics;
8. Construction of a linkage map associated with growth-related traits.

The investigated species in this Special Issue include channel catfish (*Ictalurus punctatus*), eastern mosquitofish (*Gambusia holbrooki*), Japanese flounder (*Paralichthys olivaceus*), crab (*Scylla* spp.), scallop (*Chlamys farreri*), turbot (*Scophthalmus maximus*), half-smooth tongue sole (*Cynoglossus semilaevis*), steelhead trout (*Oncorhynchus mykiss*), *Megalobrama* spp., sea cucumber (*Apostichopus japonicus*), and black rockfish (*Sebastes schlegelii*).

One of the main goals of this Special Issue is to show how transgenesis and gene/genome editing could be used to change genes or traits and help genetic improvement programs in the aquaculture industry. In particular, Qin et al. [1] reported that channel catfish (cf) were sterilized by double electroporation of TALEN plasmids targeting the cfGnRH gene. Base deletion, substitution and insertion mutations were found in sequenced cfGnRH fish, with 52.9% of P_1_ fish possessing the mutations. P_1_ mutants had lower spawning rates (20.0–50.0%) when hormone therapy was denied compared to the controls (66.7%), as well as lower mean egg hatch rates (2.0% vs. 32.3–74.3%), except for one cfGnRH-mutated female with a 66.0% hatch rate. LHRHa hormone therapy resulted in good spawning and hatch rates for mutants (*p* > 0.05). No foreign DNA sequences were found in mutant P_1_ fish, indicating no plasmid integration. After knocking out the reproductive genes in P_1_ fish, mutant and control growth, survival and appearance were similar. This transgenic sterilization technique could reversibly and reproductively restrict gene-engineered, domestic and invasive fish, thus limiting the gene flow into the natural environment.

Some gonochoristic fish have features analogous to their opposite sex, implying plasticity resembling hermaphrodites. Two adult stages of female eastern mosquitofish (*G. holbrooki*) were fed 17-methyltestosterone-containing feed (0–200 mg/kg diet) for 50 days to explore its potential for sex reversal [2]. The hormone (especially at 50 mg/kg diet)-activated the male secondary sexual organs and testicular tissues, elevated anti-Müllerian hormone gene expression and changed the females’ behaviors. This research demonstrated, for the first time, that sex reversal could be achieved in adult fish. *G. holbrooki* preserve the plasticity for sex reversal after maturity, comparable to hermaphroditic fish, and can be used to study such mechanisms in a controlled manner. We anticipate that the species’ distinctive reproductive behaviors may make it a suitable companion for zebrafish (*Danio rerio*) and medaka (*Oryzias latipes*), as well as a potential model for controlling invading pests, among other applications.

Aquaculture relies heavily on sex as an essential trait, much like it does in biological science. Li et al. [3] discovered critical miRNAs during sex differentiation in the testes and ovaries of *C. farreri* (cfa). Using a dual-luciferase reporter (DLR) assay, the researchers found that cfa-novel miR65 and cfa-miR-87a-3p_1 inhibited the expression of the female-critical gene, *foxl2*, and the male-critical gene, *klf4*. Fan et al. [4] from the same research team investigated the role of *foxl2* in oogenesis and the maintenance of ovarian function in further detail. The DLR assay, designed to assess transcriptional factors from the yeast one-hybrid (Y1H) library, was utilized to validate the promoter sequence. Upon the evidence of Y1H and expression changes between the ovaries and testes, eleven putative factors, including five unannotated factors, were discovered and confirmed to be involved in the transcriptional control of *foxl2* by RT-PCR and DLR. Their research improved our understanding of *foxl2*’s precise role in normal ovarian function.

With the advancements in sequencing technology and bioinformatics, it is exciting to investigate the function and importance of different types of molecules, such as circular RNAs (circRNAs), which govern numerous biological functions. Li et al. [5] undertook a genome-wide investigation of circRNAs in tongue sole tissues over three ovarian developmental periods and found an abundance of circRNAs in the brain, pituitary gland, ovary, and liver. Some differentially expressed (DE) circRNA parental genes were highly associated with crucial signaling pathways and may be required for ovarian development and maturation. These findings implied that circRNAs are prevalent in the production-related tissues of the tongue sole and may control ovarian maturation. 

Cheng et al. [6] established lncRNA–miRNA–mRNA interactions between gynogenetic female ovaries and sex-reversed neo-male testes in Japanese flounder (*P. olivaceus*). They discovered 6772 DE-mRNAs, 2284 DE-lncRNAs, and 244 DE-miRNAs. During the spermatogenesis of the neo-male *P. olivaceus*, a large number of steroidogenesis- and spermatogenesis-related genes and non-coding RNAs (ncRNAs) were identified, which may be modulated by let-7/miR-125b clusters and have significant functions. Both *cyp11a* and *esr2b*, essential genes in the steroidogenesis pathway, were suppressed by *let-7* and *miR-125b*. This study advanced our understanding of ncRNA dynamics in teleost gynogenesis by studying the interactions among mRNA, miRNA, and lncRNA.

Alternative splicing (AS) is an important mechanism for post-transcriptional control in organisms. Tian et al. [7] used 18 RNA-Seq datasets to assess the potential involvement of AS in the liver tissues of three euryhaline teleosts: turbot (*S. maximus*), tongue sole (*C. semilaevis*), and steelhead trout (*O. mykiss*) in response to varying salinity environments. Their findings revealed that different salinity conditions altered the splicing patterns of several RNA splicing factors, which may regulate a large number of target genes. This study highlighted the importance of AS events in the salinity adaptation of teleosts. In addition, Ye et al. [8] used single-molecule real-time (SMRT) sequencing to reconstruct a high-quality transcriptome to analyze eyestalk displacement in some F_1_ offspring of the interspecific hybrid crab (*S. serrata* ♀ × *S. paramamosain* ♂). There were 37 differentially alternative splicing (DAS) events (17 up-regulated and 20 down-regulated) and 1475 differentially expressed transcripts (DETs) in hybrid crabs with relocated eyestalks. BiP and leucine-rich repeat protein lrrA-like isoform X2 were the most significant DAS events and DETs. Five primary gene ontology terms were associated with cuticle or chitin. This study identified the genetic mechanisms behind eyestalk displacement in mud crab (*Scylla* spp.) hybridization.

Identifying the interaction between fish and external pathogenic bacteria will promote the future selection and breeding of disease-resistant fish, the invention of therapeutics, and the development of sustainable aquaculture. Xue et al. [9] developed a network of ncRNAs and mRNAs in the spleen of turbot infected with *Aeromonas salmonicida*. Four infected groups (sampled at 6, 12, 24, and 96 h post-infection) had significantly more DE-circRNAs, DE-miRNAs, and DE-mRNAs than the untreated control group. DE-mRNAs and DE-ncRNAs were enriched in immune-related pathways. The results of qRT-PCR validated the high-throughput sequencing data. Their findings demonstrated that ncRNAs regulate the expression of immune-related genes via the circRNA–miRNA–mRNA regulatory network and contribute to the immunological response of the turbot spleen in response to pathogenic microbe infection. Zhang et al. [10] used Illumina shotgun sequencing, SMRT sequencing, 10× genomics, and high-throughput chromosome conformation capture (Hi-C) technologies to reconstruct the genome of *S. schlegelii*. They systematically identified and categorized the antimicrobial peptide (AMP) genes in *S. schlegelii*. The expression of intestinal AMP was studied further in nine healthy tissues and at various infection times. They concluded that as the infection progressed, an increasing number of AMPs responded to the *Edwardsiella tarda* infection. Identification of the AMPs based on the entire genome could give a comprehensive collection of prospective AMPs, support the evaluation of immune responses to *E. tarda* infection in *S. schlegelii*, and enable the development of novel therapeutic AMPs for aquaculture intervention.

With climate change and the rise in aquaculture production, hypoxia in aquatic systems is becoming a serious environmental concern. Li et al. [11] showed that the Japanese flounder might alter its gill microstructures and induce apoptosis to lower the risk of hypoxic stress. Combining the evidence from transcription and epigenetic investigations, they found that hypoxia could activate the *EPAS1/Bad* signal pathway to induce gill apoptosis. In a second hypoxia report from the same research group, Liu et al. [12] addressed the physiological alterations in blood physiology (RBC, HGB, WBC), biochemistry (LDH, ALP, ALT, GLU, TC, TG, ALB), and hormone (cortisol) parameters. A second hypoxia-responsive *HIF-1/LDH-A* pathway in Japanese flounder was characterized at the epigenetic and transcriptional levels. Their findings together would aid in the comprehension of environmental hypoxic stress and give a solid background for the production of healthy Japanese flounder.

Genetic linkage mapping attempts to arrange genes in any linear or relative order. Genetic and genomic research depends heavily on genetic linkage maps. Recent advances in sequencing and genotyping technologies have enabled the generation of a genetic linkage map with a high degree of density and precision. Using genotyping-by-sequencing (GBS), Cui et al. [13] sequenced 132 *A. japonicus* individuals (two parents and 130 offspring). The consensus map was 3181.54 cM in length with a genetic distance of 0.52 cM. A total of 6144 SNPs were represented in 22 linkage groupings (LGs). There was a strong correlation between growth-related characteristics, including body weight, body length, and the number of papillae. One of the genes, protein still life, isoform C/SIF type 2 (*sif*), was identified in LG18 and was substantially expressed throughout larval development. Although additional research is required to determine the precise regulatory implications of the SIF protein on the development and growth of *A. japonicus* embryos, these findings provide valuable genomic resources for the selection and breeding of superior sea cucumbers with enhanced production attributes.

In recent years, environmental deterioration and human factors have decreased *Megalobrama*’s genetic diversity. Chen et al. [14] developed a whole genome database of *Megalobrama* populations using whole genome re-sequencing, analyzed population genetic structure, and inferred comprehensive evolutionary relationships through principal component analysis and population structure analysis. The whole-genome selective sweep study of populations of *Megalobrama* demonstrated the genetic basis for their adaptability to the changing ecological conditions. This research examined the population history, genetic diversity, and environmental adaptability of the *Megalobrama*. These discoveries would facilitate the conservation and utilization of *Megalobrama* germplasm.

We are aware that this Special Issue does not cover all the anticipated aspects in a constrained time frame. We would like to thank all the contributors for their efforts in enhancing our knowledge of the numerous genetic and genomic components of aquaculture, with the ultimate objective of achieving sustainable aquaculture development.
